# Juvenile idiopathic arthritis in Harlequin ichthyosis, a rare combination or the clinical spectrum of the disease? Report of a child treated with etanercept and review of the literature

**DOI:** 10.1186/s12969-021-00571-9

**Published:** 2021-06-03

**Authors:** Francesco Baldo, Michela Brena, Simone Carbogno, Francesca Minoia, Stefani Lanni, Sophie Guez, Antonella Petaccia, Carlo Agostoni, Rolando Cimaz, Giovanni Filocamo

**Affiliations:** 1 Pediatric Rheumatology, Pediatric Medium Intensity Care Unit, Fondazione IRCCS Cà Granda, Ospedale Maggiore Policlinico, Via della Commenda, 9, 20122 Milan, Italy; 2 University of Milan, Milan, Italy; 3 Dermatology Unit, Fondazione IRCCS Ca’ Granda Ospedale Maggiore Policlinico, Milan, Italy; 4 ASST G.Pini-CTO, Milan, Italy; 5 Department of Clinical Sciences and Community Health, and RECAP-RD, University of Milan, Milan, Italy

**Keywords:** Juvenile idiopathic arthritis, Harlequin ichthyosis, Intra-articular corticosteroids injection, Etanercept

## Abstract

**Background:**

Harlequin ichthyosis (HI) is the most severe phenotype of autosomal recessive congenital ichthyosis. Juvenile Idiopathic Arthritis (JIA) represents a heterogenous group of disorders all sharing the clinical manifestation of chronic arthritis. Association of HI and chronic arthritis has been reported in few cases.

**Case presentation:**

We report the case of a child with HI who developed a severe form of chronic polyarthritis during the first years of life, treated with repeated multiple joint injections, methotrexate and etanercept with good response and without any adverse events.

**Conclusion:**

The reported case and the literature review highlighted the presence of a peculiar severe seronegative polyarthritis with early onset in a series of patients with HI, suggesting that polyarthritis may be a specific manifestation of HI, rather than a rare combination of two separate conditions.

## Introduction

Harlequin ichthyosis (HI) is the most severe phenotype of autosomal recessive congenital ichthyosis, a rare heterogeneous group of recessively inherited ichthyosis which encompasses a wide range of clinical phenotypes [1, 2]. HIis due to homozygous nonsense mutations in the ABCA12 (ATP binding cassette subfamily A member 12) gene [3]. Newborns with HI present with a distinct clinical appearance encased in a dense, armor-like skin separated by polygonal deep erythematous fissures that simulate the traditional costume of a harlequin. Facial features are distorted by severe ectropion, eclabium, flattened nose, and rudimentary ears [1, 4, 5]. The skin rigidity can restrict respiratory movements, the hands and feet are ischemic, often with associated poor developed digits with claw-like appearance and osseous reabsorption; flexion deformity of the limb joints is common. Developmental delay is frequently described as well [6]. Skin barrier function is markedly impaired, which can lead to hypernatremic dehydration, impaired thermoregulation, increased metabolic demands, and increased risk of respiratory dysfunction and infection, which may cause premature death within the first days to weeks of life [7]. Historically, infants with HI did not survive beyond the neonatal period; however, prolonged survival has been achieved by intensive supportive measures, emollients and, in some cases, oral administration of systemic retinoids. The cuirass of the survivors fades within 2–3 months and they subsequently develop an erythematous, scaly, very severe ichthyotic pattern with ectropion, abnormal external ears and alopecia [8]. ABCA12 encodes for the ATP-Binding Cassette A12, a membrane associated keratinocyte-specific protein whose function is to transfer specific lipids (glycosylceramides and hydroxyceramides, main components of the lipid barrier) into the lamellar granules, which are then processed and secreted into the stratum corneum (SC) to form lipid lamellae [8]. ABCA12 deficiency results in hyperkeratosis and premature terminal differentiation of keratinocytes, as well as lack of desquamation of the corneocytes, due to transport defects of specific proteases, such as callicrein 5 and cathepsin D [9–12]. The type of ABCA12 mutation has a major impact on the severity of the disease, which is due to homozygous nonsense mutations with absent or minimal residual ABCA12 function [3]. Juvenile Idiopathic Arthritis (JIA) is the most common chronic rheumatic disease of childhood. It represents a heterogeneous group of disorders all sharing the clinical manifestation of chronic arthritis with onset before the age of 16 [13]. Different disease subtypes are classically recognized, including systemic arthritis, in which systemic manifestation are present, and other forms that are mainly characterized by joint involvement (oligoarticular and polyarticular forms), to spondyloarthropathy-like forms (enthesitis-related arthritis and psoriatic arthritis) [14]. Patients with positivity for anti-nuclear antibodies (ANA) are at higher risk to develop chronic anterior non-infectious uveitis [15]. Nonsteroidal anti-inflammatory drugs (NSAIDs), intra-articular corticosteroids injections (IACI), systemic steroids and conventional and biologic disease-modifying antirheumatic drugs (DMARDs) are well-known medications used to treat different subtypes of JIA [16, 17].

.Due to the rare nature of both diseases, association of HI and chronic arthritis has been reported in few other cases. We report the case of a child born with HI who developed a severe form of chronic polyarthritis from 4 years of age. Furthermore, we offer a literature review on this topic.

## Case report

A 7-year-old boy with HI (homozygous mutation c.541 C > T in ABCA12 gene) presented to our pediatric rheumatology clinic due to pain, swelling and joint stiffness. In the first years of life, he suffered from recurrent sepsis requiring intravenous antimicrobial treatment, caused by different bacteria such as *Staphylococcus aureus* and Stenotrophomonas maltophilia. His development was characterized by a severe psychomotor delay and required an intensive multidisciplinary follow-up. The child was on treatment with systemic retinoids from birth.

**Fig. 1:**
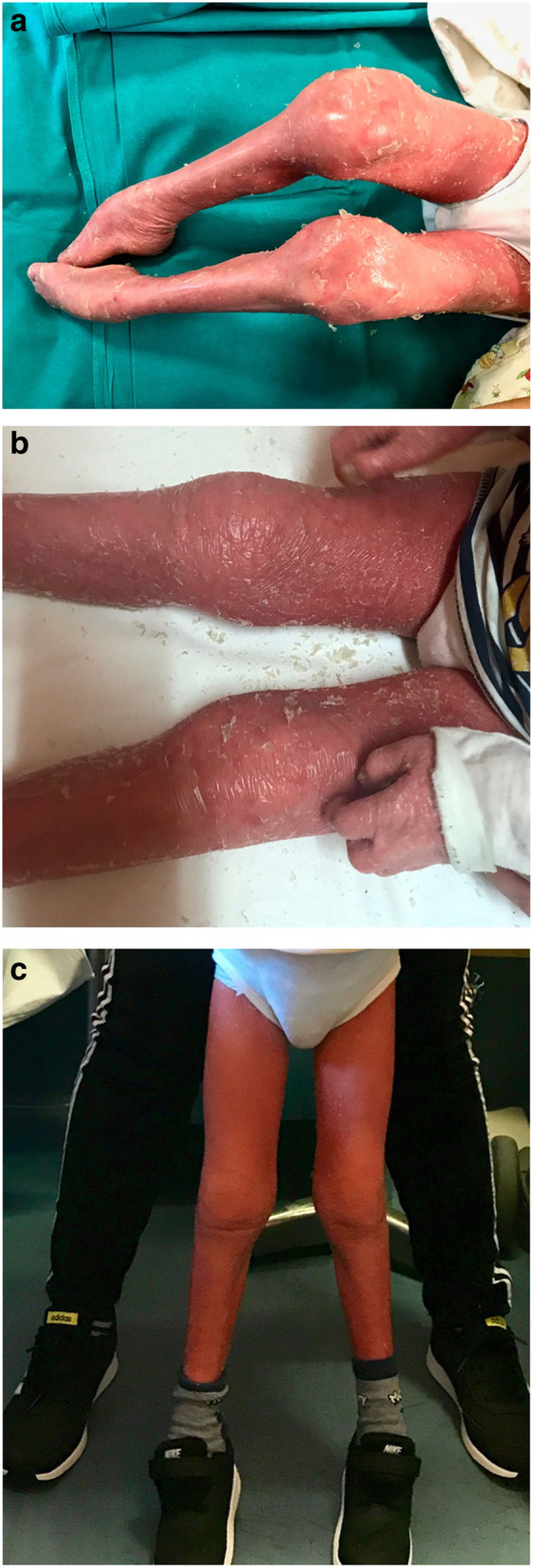
a-b-c: Severe arthritis in a 4 years old boy affected by Harlequin Ichthyosis. a. At visit 0 in our Clinic. b. After 2 months from the first multiple intraarticular steroids injection. c. After 8 months from the beginning of etanercept

The mother reported a 3 year-history of chronic polyarthritis. At disease onset, both knees showed swelling and the child developed inability to walk. He was then unsuccessfully treated with antimicrobial therapy in suspicion of septic arthritis. Subsequently all the large joints were involved, causing severe limitation on motion, particularly of both ankles. At the time of the examination, he had flexion contractures of fingers and of all large joints of the lower limbs (Fig. 1a). Ultrasonographic (US) evaluation showed marked synovial effusion in both knees, synovial hypertrophy with increased Doppler signal in both ankles and wrists and no evidence of tenosinovitys. X-rays of the affected joints was unremarkable, except for decresed bone density. Laboratory investigations were normal except for the presence of microcytic anemia and a mild increase in C-reactive protein (CRP). ANA, anti-extractable nuclear antigen (ENA), anti-cyclic citrullinated peptide (CCP) antibodies and rheumatoid factor (RF) were negative. An ophthalmology examination did not show uveitis. According to the International League of Associations for Rheumatology (ILAR) classification [18], the clinical features were consistent with polyarticular RF negative JIA. The boy underwent multiple IACI (triamcinolone hexacetonide, for large joints and methylprednisolone acetate for small joints). Synovial fluid was turbid, whit a white cell count of a 750/mm^3^ count. A second line treatment with oral methotrexate (15 mg/m^2^ weekly) was also started. (Fig. 1b).

Three months later, owing to the presence of persistently active disease in the ankles, IACI were repeated. Despite this treatment, a complete remission of arthritis was not achieved. Therefore, 8 months after the beginning of methotrexate, a third IACI was performed and a Tumor Necrosis Factor-alpha (TNFα) antagonist was started (etanercept 0,8 mg/kg weekly). In 2 months a status of minimal disease activity (absence swollen joint, and a parent and physician global assessment of disease activity of 3 and 2 cm on a VAS scale, respectively) was reached [19]. Appropriate physical therapy was started, and articular functional ability improved in the following months, allowing the child to walk again. Improvement of skin erythema was observed as well.

Eighteen months after beginning etanercept treatment, the patient was stable in minimal disease activity with a further improvement in motor functional abilities and no medication side effects were reported (Fig. 1c). Methotrexate has been recently tapered to every other week administration.

## Case review

Development of JIA in HI patients has already been described. Information about family history, genetics and treatment of the different cases is shown in Table 1. The first case of HI and arthritis was reported by Chan et al. [23], who described a female newborn of 2250 g, delivered at 37 weeks of gestational age who showed classical HI appearance at birth and was admitted to neonatal intensive care unit (NICU), treated with intravenous and topical antibiotics for skin infections. Oral etretinate at a dose of 1 mg/kg/day was initiated at the age of 36 days (replaced with acitretin at 7 years of age). At 6 years, she developed swelling of the wrists and ankles as well as of small joints of the hands, with radiologic evidence of erosive arthritis. She received prednisolone, and ibuprofen at a dose of 30 mg/kg till she was 11 years of age, when it was replaced by rofecoxib. At 9 years of age, because of inadequate disease control, methotrexate (MTX) was added, with an improvement of erythroderma as reported by parents. Auriti et al. described two other cases [20]. The first one was male, delivered via caesarean section (CS) at 35 weeks because of preterm prelabour rupture of membranes (pPROM), with a birth weight of 2300 g and admitted to NICU. Retinoid treatment was started in the first day of life and stopped after 10 days. During his early life, he needed multiple red blood cells and platelet transfusions due to severe anemia, and several intravenous antibiotic courses for recurrent sepsis. At 3 months of life, he developed severe knee swelling, which was, the first time, thought to be due to septic arthritis, but never completely resolved after antibiotic treatment. Laboratory tests showed mild increase in CRP and erythrocyte sedimentation rate (ESR); both ANA and RF were negative. He presented multiple flares of knee arthritis, unsuccessfully treated with oral NSAIDs. At the age of two he underwent IACI, started physiotherapy and achieved complete remission.

**Table 1:** Summary of genetic results and treatment reported in cases of JIA associated with HI

	Parents	Genetics	Treatment
**Auriti et al, (2020) Case 1** [20]	unrelated	compoud heterozygosity c.4036delG and c.7444C > T genomic variants in the *ABCA12* gene	NSAIDs (ineffective); intra articular triamcinolone hexacetonide 1 mg/kg) at 2 years
**Auriti et al, (2020) Case 2** [20]	unrelated	compound heterozygosity c.224 T > A, c6610C > T, C164G > A c.346G > T in *ABCA12* gene; variant c.817G > A in heterozygosity in *TGM1* gene	Ibuprofen (effective, but relapse at discontinuation); repeated IACI and weekly MTX, at 2 months: clinical and radiological resolution of all joint except both ankles because of anterolateral compartments tendon synovitis
**Clement et al (2007)** [21]**; Rajpopat et al (2011)** [6]**; Raghuvanshi S et al (2015)** [22]	unknown	*ABCA12* mutation	NSAIDs (ineffective); Etoricoxib (temporarily effective); MTX (temporarily effective) Etanercept; total Hip arthroplasty by the age of 17* (see Rajpopat et al. Arch. Dermatol, June 2011); subsequently tried on adalimumab, MTX, then leflunomide. Reported worsening of skin condition and increased skin infections
**Rajpopat et al (2011)** [6],	unknown	Unknown	NSAIDs for 1 year
**Raghuvanshi S, et al (2015)** [22], **Rajpopat et al (2011)** [6]	unknown	Unknown	Intensive physiotherapy, splints and intra-articular injections.
**Chan et al (2003)** [23]	unrelated	Unknown	Prednisolone. Ibuprofen was started concurrently, and maintained until she was 11 years of age, then replaced by rofecoxib. MTX was added at the age of 9 years.

*Abbreviations*: *NSAIDs* Nonsteroidal anti-inflammatory drug, *IACI* Intra-Articular Corticosteroids Injection, *MTX* Methotrexate

The second case was a male, born late preterm, delivered via CS. At birth, he showed persistent bradycardia, hypotonia and generalized cyanosis, and needed a 30″ cardiopulmonary resuscitation. He was born showing collodion baby features with thickened and fissured skin, flexion contractures of upper and lower limbs and collodion like membrane all over the body. He was admitted to NICU, and developed *Enterococcus faecalis* sepsis and bronchitis. Acitretin treatment was started in early life for 1 month, then stopped because of liver toxicity. He was also given a physiotherapy rehabilitation program for congenital contractures. At 2 years of life, he developed severe hands, right knee, elbows, wrists and tibiotalar, subtalar and talonavicular joint arthritis associated with diffuse tenosynovitis of fingers, wrists, ankles. The child lost the ability to walk because of severe polyarthritis. Elevation of white blood cells, eosinophils, IgE, CRP and ESR were reported, while RF was negative. The child was first treated with ibuprofen and then received multiple IACI and weekly MTX. Clinical and radiological resolution was achieved, except for persistent tenosynovitis of both ankles.

Clement et al. described another case of a term male newborn with classical HI appearance at birth, who received treatment with acitretin. At 10 years, he developed severe arthritis in multiple joints. Blood tests showed microcytic hypochromic anemia and raised CRP levels; ENA, anti-double stranded DNA and RF were negative. Initially, he received NSAIDs without clinical improvement, then he was shifted to Etoricoxib with temporary response. The patient was then given MTX and Etanercept at 25 mg/week with improvement in mobility, but he needed total hip arthroplasty by the age of 17. Etanercept was then discontinued, probably because of worsened skin condition [21] and the patient was switched to adalimumab, MTX and leflunomide. However, worsening of the cutaneous disease with cracks and increased skin infections was reported with all previous DMARDs and biologics [6, 22].

In a case series of 45 patients with molecular diagnosis of HI, Rajpopat et al. [6] report two more cases of children with signs of arthritis: one showed bone erosions on radiographs, the other intermittent swelling of the knee. The first has probably more recently been described also by Raghuvanushi et al. [22]: the patient was referred to the musculoskeletal service because of a long-term condition of painful elbows, shoulders, fingers, and locking of wrists. Conventional radiography documented destructive alterations of wrists with subluxation of proximal row of carpal bones. A magnetic resonance imaging (MRI) scan of shoulders revealed rotator cuff tendinopathy and small joint effusion without synovitis. The patient was seronegative and is managed with IACI and physical therapy rehabilitation. No further details are available for the second case.

## Discussion

The association of HI and arthritis has been reported in literature, however, to date few cases have been described. It is unclear whether the concomitant arthritis should be regarded as the manifestation of a concomitant JIA or whether it represents a specific entity with unique features of HI itself. The longer survival of HI patients may increase the number of patients who may develop arthritis throughout the disease course, and will increase the probability to better understand in the future the best management of joint involvement in the published cases, arthritis showed a rather aggressive course, leading to joint erosions and even to early joint replacement. Most patients had early arthritis onset. Acute phase reactants were usually only slightly increased, while ANA, FR and ENA were found to be negative. Response to IACI was generally good, although temporary, and response to conventional and biologic DMARS was variable.

In JIA immune cells, including T and B lymphocytes, infiltrate the synovial membrane of inflamed joints, suggesting that the adaptive immune system is involved in the pathogenesis of the disease [24]. Recently, a particular T lymphocyte population, Th17 cells, has been found to be crucial in JIA pathogenesis: TNF-α inhibitors are the main biological drugs used in JIA and interfere with these cells, giving an explanation of efficacy of etanercept treatment in JIA [25].

Harlequin Ichthyosis is characterized by a profound dysregulation of lipid secretion into the stratum corneum and skin barrier function. Currently, therapy is focused on the treatment of scaling, using keratolytics such as urea [26], or long-term retinoids [27], which may negatively affect bone density [28], and can generate skin erosion and thinning leading to further epidermal barrier function impairment [29]. Retinoids have also been shown to cause arthropathy, but this typically presents in the axial skeleton as ossification along the anterior longitudinal ligament and as pelvic hyperostosis.

As already found in other dermatoses such as atopic dermatitis and psoriasis, cytokine dysregulation and barrier impairment are both factors that underpin the disease [30]. In different types of ichthyosis, high levels of IL-17 and TNF-α were documented [31, 32]. This is accompanied by increased systemic and skin-homing T-cell activation and multicytokine polarization, with IL-17/IL-22 polarization predominance [33]. Ustekinumab, a monoclonal antibody directed against IL-23, was able to reduce skin erythema, scaling, and Trans Epidermal Water Loss (TEWL), a skin barrier functional index, in two patients with an ichthyotic syndrome [31]. To investigate the therapeutic potential of IL-17 targeting drugs, a clinical trial of secukinumab (anti-IL17 antibody) in patients with ichthyoses (NCT03041038), is currently ongoing. Concurrently, efficacy of secukinumab is being studied in enthesitis-related arthritis and psoriatic arthritis, two different JIA subtypes (NCT03031782). Both treatments, acting on IL-17/IL-23 pathways at cutaneous and articular level, could represent an important therapeutical option in those patients who suffer from HI and arthritis.

Cutaneous and systemic immune inflammation has been well studied and successfully treated in other skin diseases such as psoriasis and atopic dermatitis [33]. However, inflammation in ichthyosis is little considered. In psoriasis, biologic treatment with IL-17 antagonist is reported as highly effective in reversing the inflammation and cutaneous disease [31]. Of interest, the role of anti- TNF-α treatment in psoriasis and psoriatic arthritis is already well documented [34].

HI patients benefit from retinoid therapy. A role of retinoic acid in modulating IL-17 pathway and TH-17 differentiation has been pointed out. Of interest, retinoic acid was proven to promote T naïve differentiation in regulatory T cells and inhibits the differentiation of naive T cells into Th17 cells. Surprisingly, in vitro and animal studies suggested that in an inflammatory context, retinoic acid stimulates effector T cell responses during infection or autoimmune diseases [35].

Conventional DMARDs in patients with HI and chronic inflammatory arthritis have already been used without significant side effects. In a single case report worsening of erythroderma and increased cutaneous infections were reported, with poor disease control even using MTX and a biologic agent in combination [21]. In our patient, the severity of arthritis required the association of conventional and biological DMARDs with improvement of arthritis and decrease of erythema, without any infectious events reported at 18 months follow-up, nor any other adverse events.

The role of other proinflammatory cytokines and other biology treatments in ichthyosis have been proposed. Interleukin (IL)1 alpha is constitutively expressed in the upper epidermis [36, 37], as is its receptor [38], and the soluble decoy receptor (IL1RA) [39, 40], and a fine balance in expression of IL1 alpha and IL1RA is a prerequisite for healthy skin [41]. Some studies suggest that up-regulation of IL-1 is common to all Autosomal Recessive Congenital Ichthyoses, and the entity of this upregulation is related to clinical severity. An in vitro study on disease-mimic organotypic cultures treated with IL-1 receptor antagonist was beneficial on hyperkeratosis in a dose-dependent fashion [42].

JIA is now thought to be a multifactorial pathology, in which genetic susceptibility meets an environmental trigger, leading to an uncontrolled response toward putative self-antigens [43]. Some of the environmental triggers that have been studied as risk factors for the development of arthritis are frequently reported in HI patients [44]. Patients with HI often have an history of preterm birth and caesarean section delivery, and undergo extensive use of antibiotics in early life. Antibiotic use was associated with JIA development in a large pediatric population, and could play a role in JIA pathogenesis by acting through microbiome disruption [45]. Some studies have proposed that unlabored C-sections may slightly increase the risk of JIA due to changes in newborn microbiota and immune response [46, 47].

## Conclusion

Few cases of HI have been reported and survival through early life is still a hard challenge for families and clinicians alike. However, with reducing in early mortality of patients with HI, further disease related complications, leading to a decrease in patients’ quality of life may become more frequent.

In our patient HI has been found to be associated with highly aggressive arthritis, in line with the cases previously reported in literature. The articular involvement in HI seems to have specific features and may need a different therapeutic approach from classic JIA.

Although the exact pathogenetic mechanism underlying HI-associate arthritis is still to be clarified, a prominent role of inflammation has been demonstrated in HI.

This can also drive the choice of a common therapeutic target for both arthritis and some features of the cutaneous disease in patients with HI itself. No resolutive therapy exists, to date, for HI, even if experimental gene therapies are in preclinical development [48]. Together with conventional therapies, a role of anti-inflammatory drugs, and especially of biologic therapy in patients with severe congenital forms of ichthyosis may deserve investigation.

## Data Availability

The datasets used during the current study and the informed consent for publication are available from the corresponding author on reasonable request.

## Abbreviations

ANA: Anti-nuclear antibodies
CCP: Cyclic citrullinated peptide
CRP: C Reactive Proteine
CS: Caesarean section
DMARDs: Disease-modifying antirheumatic drugs
ENA: Anti-extractable nuclear antigen
ESR: Erythrocyte sedimentation rate
HI: Harlequin ichthyosis
IACI: Intra-articular corticosteroids injections
IL: Interleukine
ILAR: International League of Associations for Rheumatology
JIA: Juvenile Idiopathic Arthritis
MRI: Magnetic resonance imaging
MTX: Methotrexate
NICU: Neonatal intensive care unit
NSAIDs: Nonsteroidal anti-inflammatory drugs
pPROM: Preterm prelabour rupture of membranes
RF: Rheumatoid factor
SC: Stratum corneum
