# Correction to: Juvenile idiopathic arthritis in Harlequin ichthyosis, a rare combination or the clinical spectrum of the disease? Report of a child treated with etanercept and review of the literature

**DOI:** 10.1186/s12969-021-00603-4

**Published:** 2021-07-23

**Authors:** Francesco Baldo, Michela Brena, Simone Carbogno, Francesca Minoia, Stefano Lanni, Sophie Guez, Antonella Petaccia, Carlo Agostoni, Rolando Cimaz, Giovanni Filocamo

**Affiliations:** 1 Pediatric Rheumatology, Fondazione IRCCS Ca’ Granda Ospedale Maggiore Policlinico, Milan, Italy; 2 University of Milan, Milan, Italy; 3 Dermatology Unit, Fondazione IRCCS Ca’ Granda Ospedale Maggiore Policlinico, Milan, Italy; 4 ASST G.Pini-CTO, Milan, Italy; 5 Department of Clinical Sciences and Community Health, and RECAP-RD, University of Milan, Milan, Italy

**Correction to: Pediatr Rheumatol 19, 80 (2021)**

**https://doi.org/10.1186/s12969-021-00571-9**

Following publication of the original article [1], the authors identified an error in the author name of Stefano Lanni.

- The incorrect author name is: Stefani Lanni
- The correct author name is: Stefano Lanni

The author group has been updated above and the original article [1] has been corrected.
